# Hydrophobic dual-polymer–reinforced graphene composite aerogel for efficient water–oil separation†

**DOI:** 10.1039/d4ra06747a

**Published:** 2025-01-03

**Authors:** Zirong Luo, Shenbo Huang, Na Kong, Jizhen Zhang, Jinlong Tao, Jihua Li, Shuang Li

**Affiliations:** a Hainan Provincial Key Laboratory of Natural Rubber Processing, Agricultural Products Processing Research Institute, Chinese Academy of Tropical Agricultural Sciences Zhanjiang 524001 P. R. China luozirong3@163.com jinlongt1983@163.com; b Guangdong Engineering & Technology Research Centre of Graphene-like Materials and Products, Department of Chemistry, College of Chemistry and Materials Science, Jinan University Guangzhou 510632 P. R. China; c School of Life and Environmental Science, Centre for Sustainable Bioproducts, Deakin University Geelong Victoria 3216 Australia; d Institute for Frontier Materials, Deakin University Geelong Victoria 3216 Australia; e Chinese Academy of Tropical Agricultural Sciences Haikou 571101 P. R. China

## Abstract

Addressing the environmental challenges posed by oil spills and industrial wastewater is critical for sustainable development. Graphene aerogels demonstrate significant potential as highly efficient adsorbents due to their high specific surface area, excellent structural tunability and outstanding chemical stability. Among available fabrication methods, the hydrothermal self-assembly technique stands out for its low cost, high tunability and good scalability. However, brittleness caused by stacking and agglomeration of graphene layers during self-assembly remains a significant challenge. In this study, we present a green and efficient self-assembly strategy combining a one-step hydrothermal process with a solution immersion method to fabricate a PDMS-coated epoxidized natural rubber–graphene composite aerogel (P@EGA). The resulting aerogel exhibits a high specific surface area (482.362 m^2^ g^−1^), hierarchical pore distribution from microporous to macroporous, ultra-low density (0.0104 g cm^−3^) and excellent hydrophobicity (contact angle = 147.6°). Remarkably, it retains 97.54% of its compressive stress after 50 compression-release cycles at 80% strain and quickly recovers its shape under a 500 g load. The P@EGA aerogel demonstrates outstanding adsorption capacities (65.37–132.75 g g^−1^) for various oils and organic solvents, complete oil absorption in 0.4 seconds, and effortless regeneration through simple squeezing. Furthermore, its dual functionality in gravity-driven and powered water–oil separation systems underscores its broad application potential in environmental remediation.

## Abstract 2

Novel PDMS-coated epoxy natural rubber–graphene aerogels enhance flexibility, hydrophobicity, and oil adsorption for environmental applications.

## Graphical abstract

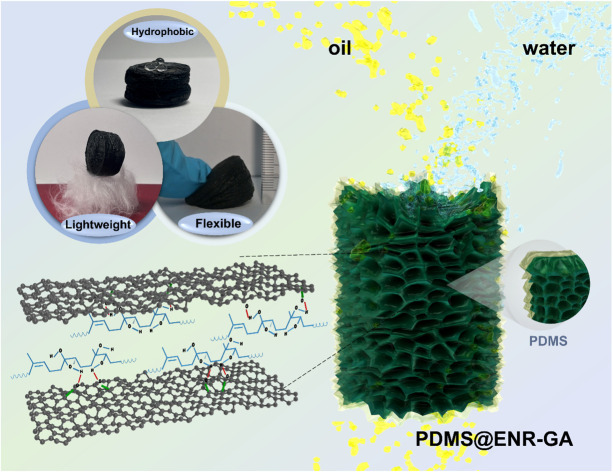

## 1 Introduction

With the increasing global industrialization and urbanization, the generation of oily wastewater from both production processes and daily activities has risen significantly. This trend poses serious risks to environmental health and biological ecosystems, potentially leading to severe ecological imbalances.^1–4^ Therefore, effective treatment of water pollution caused by oil spills and industrial wastewater has become a pressing issue for the global community. Graphene aerogels, known for their high specific surface area, environmental friendliness, excellent chemical stability, and inherent hydrophobicity, have been extensively studied for applications in sensors,^5^ supercapacitors,^6^ energy storage,^7^ and particularly in oil–water separation and oil spill remediation.^8–10^ Among the various methods for synthesizing three-dimensional (3D) graphene aerogels, the hydrothermal reaction method is considered the most promising for large-scale production due to its low cost, high tunability, and scalability.^11,12^ However, during self-assembly, graphene sheets tend to stack due to strong interactions, including van der Waals forces and π–π conjugation. This stacking significantly reduces the specific surface area and the number of active adsorption sites, leading to the inherent brittleness and low mechanical strength of the prepared aerogel, which becomes prone to fracture under mechanical stress.^13,14^

To mitigate this brittleness, flexible skeletal materials such as nanomaterials,^15,16^ polymer matrices,^17–19^ and biomass^20^ can be incorporated between graphene sheets. These materials inhibit sheet stacking and enhance the mechanical properties of the resulting composites. Polymers, in particular, show promise for fabricating elastic graphene composite aerogels. For instance, Huang *et al.* introduced a polyimide layer into graphene aerogels using a vacuum infiltration curing method, achieving a robust 3D network structure with a compressive strength of 175 kPa.^21^ Polydimethylsiloxane (PDMS) is a biocompatible and highly flexible polymer commonly utilized as a hydrophobic or elastomeric material.^22^ Wu *et al.* developed PDMS-coated 3D MXene aerogels that maintained a high electromagnetic interference (EMI) shielding efficiency of 48.2 dB after 500 compression-release cycles, demonstrating exceptional compressibility and durability.^23^ While the application of polymers on the surface of graphene aerogels can improve flexibility, achieving full penetration into the internal 3D network remains challenging, limiting the extent of toughness enhancement. Furthermore, polymers can also act as flexible supporting skeletons, blending with graphene to construct stable 3D porous networks hydrogen bonding and other interactions, addressing agglomeration, limited adsorption sites, and poor mechanical properties. For example, Liu *et al.* synthesized graphene/polypyrrole composite aerogels using a one-step hydrothermal method.^24^ The addition of polypyrrole nanorods (PNRs) not only prevented the aggregation of graphene sheets and enhanced mechanical strength but also effectively modified the dielectric constant of the aerogels. Similarly, Lu *et al.* developed graphene composite aerogels with high compression resistance and durability by introducing polyacrylic acid (PAA) during freeze casting.^25^ Notably, at approximately 30 wt% PAA, the aerogel's strength increased by 200–300%. Natural rubber is a natural polymer material characterized by excellent elasticity, abrasion resistance, and mechanical strength. Epoxidized natural rubber is produced by introducing epoxy groups *via* an epoxidation reaction, which not only preserves the high elasticity and strength of natural rubber but also enhances its polarity. These polar oxygenated groups can form stronger chemical or hydrogen bonds with the surface groups of graphene aerogel.^26,27^ This improved interfacial bonding facilitates better dispersion of graphene, thereby enhancing the mechanical properties of the composites.

In this study, we designed a comprehensive strategy using epoxidized natural rubber (ENR) particles as a flexible support skeleton, combined with graphene oxide through hydrothermal reduction self-assembly. This approach effectively weakened the π–π conjugation between graphene sheets, thereby enhancing the strength and flexibility of formed aerogels. The resulting Epoxidized Natural Rubber/Graphene Aerogels (EGA) featured a stable 3D porous network structure. To further reinforce the EGA polydimethylsiloxane (PDMS) was introduced as a secondary phase, providing additional strength and imparting hydrophobic properties. These enhancements enabled effective and continuous adsorption and separation of various oils and organic solvents.

## 2 Experiments

### 2.1 Materials

Epoxidized natural rubber latex (epoxy degree 50 mol%) was obtained from the Agricultural Products Processing Research Institute of the Chinese Academy of Tropical Agricultural Sciences. Graphite powder (99.5%) was purchased from Shanghai Yuanye Biotechnology Co., Ltd. Concentrated sulfuric acid (H_2_SO_4_), potassium permanganate (KMnO_4_), hydrogen peroxide (H_2_O_2_), and concentrated hydrochloric acid (HCl) were all procured from Guangzhou Chemical Reagent Factory. Ascorbic acid was supplied by Shanghai Aladdin Biochemical Technology Company. Polydimethylsiloxane Dow Corning 184 was purchased from Dow Corning Corporation in the United States, while Sudan Red III and Methylene Blue were obtained from Kemio Reagent Company. All chemicals used in this study were analytical grade reagents and used without further purification.

Organic solvents were purchased from Guangzhou Chemical Reagent Factory. Pump oil (density is 0.85 g cm^−3^, viscosity is 90.15 mm^2^ s^−1^) was obtained from Mitsubishi Chemical Corporation, Japan. Cooking oil (density is 0.93 g cm^−3^, viscosity is 10.25 mm^2^ s^−1^) was purchased from Wal-Mart. Diesel oil (density is 0.83 g cm^−3^, viscosity is 4.36 mm^2^ s^−1^) was supplied by Zhongke Refining & Petrochemical Co.

### 2.2 Preparation of graphene oxide

Graphene oxide (GO) was synthesized using an improved Hummers' method.^28^ Graphite powder (2 g) and sulfuric acid (H_2_SO_4_, 100 ml) were mixed in a three-necked flask and stirred for 1 hour. Potassium permanganate (KMnO_4_, 15 g) was then added gradually under ice bath conditions, maintaining the temperature below 10 °C and keep stirring for 12 h. Upon completion of the reaction, pure water and hydrogen peroxide (H_2_O_2_) were added dropwise until the solution turned yellow. The resulting mixture was centrifuged, and the sediment was washed three times with a hydrochloric acid solution (HCl : H_2_O = 1 : 9), followed by washing with pure water until the pH reached neutral.

### 2.3 Preparation of epoxidized natural rubber/graphene composite aerogel (EGA)

Epoxidized natural latex (0.4, 0.8, 1.2, and 1.6 ml, 80 mg ml^−1^) was added to a GO dispersion (20 ml, 8 mg ml^−1^) and ultrasonically dispersed for 5 min. Ascorbic acid (160 mg) was then added and the mixture was magnetically stirred for 15 min at room temperature. Keeping stirring, the suspension was sealed and placed in an oven at 180 °C for 6 h. After the reaction, the product was washed three times with deionized water, frozen at −18 °C for 12 h to form an ice template and subsequently freeze-dried at −80 °C for 24 h. This process yielded the epoxidized natural rubber–graphene aerogel (EGA). The aerogel samples were designated as EGA_20%_, EGA_40%_, EGA_60%_, and EGA_80%_, based on the latex-to-GO ratio. For comparison, a pure graphene aerogel (GA) was prepared using the same method, excluding the addition of epoxidized natural latex.

### 2.4 Preparation of hydrophobic layer composite aerogel

Dow Corning 184DC and its curing agent were weighed in a 10 : 1 ratio and dissolved in ethyl acetate. The EGA was immersed in this solution for 30 min, then dried at 60 °C to remove the ethyl acetate. The aerogel was further cured in a vacuum oven at 120 °C for 10 h, resulting in a PDMS-coated epoxidized natural rubber–graphene aerogel (P@EGA).

### 2.5 Characterizations

The microstructure of the aerogels was examined using a field emission scanning electron microscope (SEM, HITACHI-S4800) operated at 5 kV. Elemental distribution in the hydrophobic samples was analyzed using a Bruker elemental analysis system. Zeta potential measurements were conducted using a Zeta Potentiometer (Malvern Panalytical Zetasizer Pro) after ultrasonic pretreatment of the GO dispersion and ENR latex. Fourier transform infrared spectra (FT-IR) were recorded using a Bruker Vertex 70 spectrometer in attenuated total reflection (ATR) mode over a range of 600–4000 cm^−1^. X-ray diffraction (XRD) was was obtained with a D/max-1200 diffractometer (Cu Kα radiation at a scanning speed of 4° min^−1^ and a range of 5–40°). Raman analysis was performed with a confocal Raman microscope (CRM, Alpha300R, WITec GmbH) equipped with 532 nm (40 mW, WITec GmbH). Surface composition analysis was performed using X-ray photoelectron spectroscopy (XPS, Thermo Scientific K-Alpha). The specific surface area and pore size distribution of the aerogels were measured *via* nitrogen adsorption–desorption at 77 K using an automatic specific surface area and porosity analyzer (Micromeritics ASAP 2460). The contact angle of the aerogels was measured with a contact angle analyzer (SL200 B, American Corona Industry Co., Ltd), with the final result representing the average of three tests. Mechanical performance data were obtained using a Tensile Tester TM2101-T5, with a load capacity of 1 kN and loading/unloading speeds of 50 mm min^−1^.

### 2.6 Adsorption experiments

To assess adsorption properties, the aerogel was immersed in various oil products and organic solvents until adsorption equilibrium was reached. After equilibrium, the samples were weighed after ensuring no additional liquid droplets remained on the surface. The adsorption capacity (*Q*) was calculated using the following formula:

1 *Q* = (*m*_s_ − *m*_0_)/*m*_0_

where *m*_0_ is the initial weight of the sample and *m*_s_ is the weight of the sample after reaching adsorption equilibrium.

The recycling performance of the aerogels was evaluated using squeezing and extraction method. After reaching adsorption equilibrium, the aerogels were subjected to external force to squeeze out the adsorbed liquid or treated with *n*-hexane to extract all adsorbates. After this treatment, the aerogels were weighed again. This process was repeated over a total of 10 cycles. Each experiment was independently replicated three times, and the average results were reported to ensure reliability.

## 3 Results and discussion

### 3.1 Preparation and microstructure characterization of composite aerogels

**Fig. 1:**
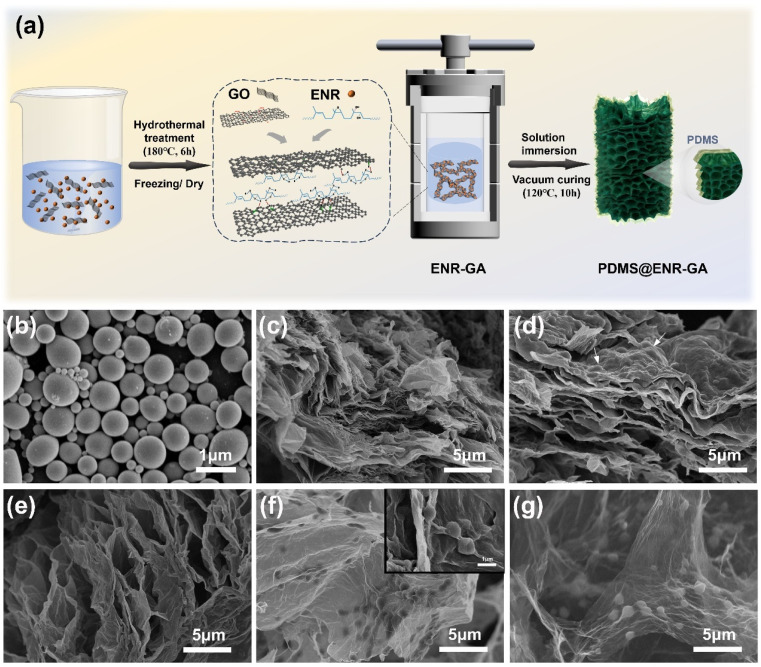
(a) Schematic diagram of preparation process of P@EGA, SEM images of (b) ENR particles, (c) GA, (d–f) EGA, (g) P@EGA.

The synthesis diagram of the P@EGA is presented in Fig. 1a. A stable dispersion of graphene oxide (GO) with a zeta potential of *δ* = −37.06 mV was mixed with epoxidized natural rubber (ENR) latex, which had a zeta potential of *δ* = −48.55 mV. During the hydrothermal reaction, the epoxy groups in ENR underwent ring-opening, generating hydroxyl groups that formed hydrogen bonds with the oxygen functional groups on GO. These interactions facilitated the embedding of ENR within the graphene sheets, weakened the π–π stacking between them and promoted the assembly of a stable 3D porous network structure. This process also partially reduced the GO.

Subsequently, a polydimethylsiloxane (PDMS) layer was successfully coated between the sheets using the solution immersion method, resulting in a lightweight P@EGA with excellent hydrophobic properties. To examine the microstructure (Fig. 1b), ENR particles were fixed with osmium acid staining, revealing particle diameters ranging from 0.2 and 1.2 μm. As shown in Fig. 1c, the 3D structure of the pure GA consists of graphene sheets with significant stacking between layers. In contrast, Fig. 1d–f clearly illustrate that the graphene sheets in the EGA are covered with bumps corresponding to the ENR particles, confirming their successful embedding. This arrangement generates layered porous folds and numerous channels. A comparative analysis of aerogels with varying ENR additions revealed that EGA_40%_ exhibits a more pronounced opening geometry and a superior pore structure compared to EGA_20%_. However, with increased ENR proportions, the intermolecular forces among ENR particles exceed the hydrogen bonding forces between graphene sheets, leading to a more disordered structure. At an 80% ENR-to-GO ratio, stacking and fusion of ENR particles were observed. Graphene sheets were sporadically stacked on the particle surfaces, leading to a significant reduction in the channels within the aerogel (ESI, Fig. S1†).

**Fig. 2:**
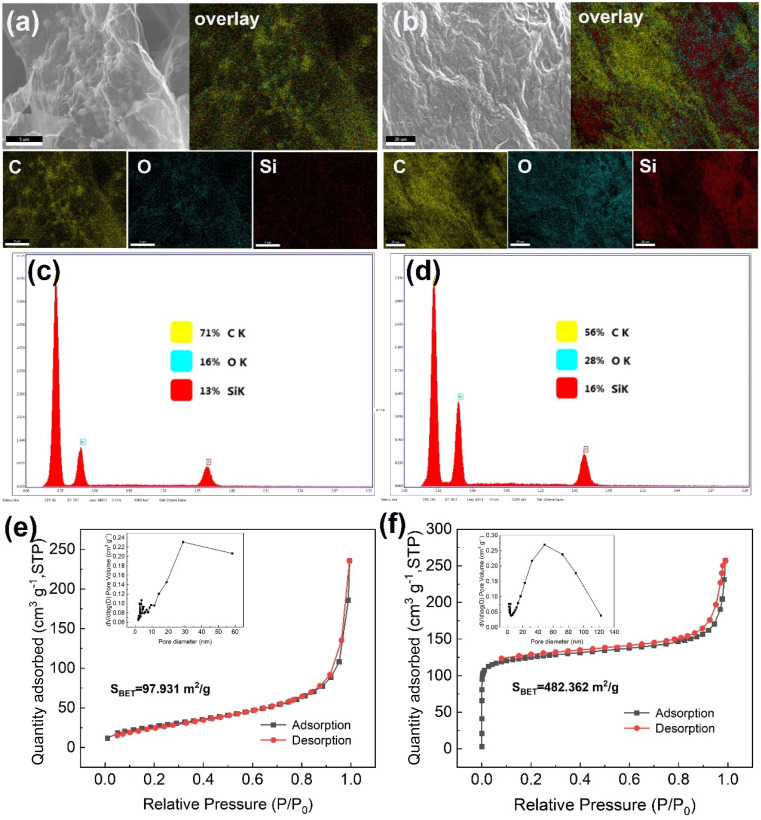
Elemental distribution mapping of (a) internal surface and (b) external surface of P@EGA; EDS spectra of (c) internal surface and (d) external surface of P@EGA; N_2_ adsorption–desorption isotherms and the pore-size distribution curves of (e) GA, (f) P@EGA.

As shown in Fig. 1g and ESI S2,† the graphene sheets within the P@EGA remained well-dispersed, with no adhesion observed between the sheets. This demonstrates that the PDMS coating adhered solely to the surface of the walls and did not influence its 3D structure. Energy dispersive spectroscopy (EDS) elemental mapping (Fig. 2a and b) confirms that the predominant elements on both the inner and outer surfaces of the P@EGA are carbon (C), oxygen (O), and silicon (Si), validating the successful PDMS coating on the composite aerogel. As illustrated in Fig. 2c and d, the distribution ratio of oxygen and silicon on the outer surface is slightly higher than on the inner surface. This indicates that the PDMS has penetrated the 3D network structure, although the penetration is incomplete and uneven. As shown in Fig. 2e and f, the adsorption curves of GA and P@EGA exhibited hysteresis loops characteristic of capillary condensation, aligning with type IV isotherms according to IUPAC classification.^29^ Notably, the BET specific surface area of P@EGA (482.362 m^2^ g^−1^) was significantly higher than that of GA (97.931 m^2^ g^−1^). Additionally, the adsorption curve of P@EGA showed a rapid increase in adsorption capacity at low *p*/*p*_0_ region, attributed to micropore volume filling, indicating the presence of both micropores and mesopores. Compared to GA, the composite aerogels exhibited a broad pore size distribution, ranging from several nanometers to hundreds of nanometers, retaining some larger pores. This hierarchical pore structure, enriched by the incorporation of ENR particles, formed a stable 3D network. The micropores present in the P@EGA contributed to a higher specific surface area and more active sites, while mesopores facilitated the rapid diffusion of adsorbates, enhancing the adsorption performance for oil spills and organic pollutants.

### 3.2 Physicochemical characterization of composite aerogels

**Fig. 3:**
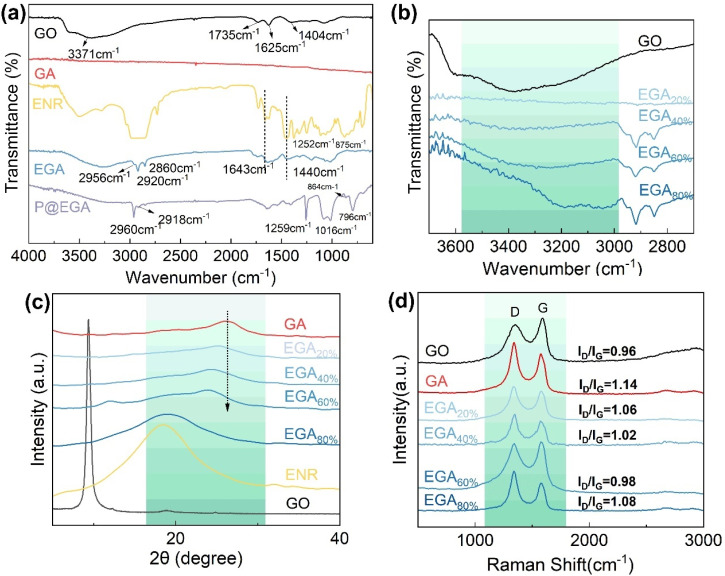
(a and b) FTIR spectra of GO, GA, ENR, EGA and P@EGA, (c) XRD patterns of GO, GA, ENR and EGA, (d) Raman spectra of GO, GA and EGA.

The FT-IR spectra of GO, GA, ENR, EGA, and P@EGA are presented in Fig. 3a. The GO spectrum displays peaks at 3371 cm^−1^, 1735 cm^−1^, 1625 cm^−1^, 1404 cm^−1^ correspond to C–OH stretching vibration, C=O peaks of ketones and carboxyl groups, the C=C stretching vibration peak, and C–OH stretching of hydroxyl groups, respectively. These peaks confirm the presence of abundant oxygen-containing groups.^30^ In contrast, these characteristic peaks are nearly absent in pure GA, suggesting substantial reduction of GO during hydrothermal treatment. For ENR, peaks at 1249 cm^−1^ and 875 cm^−1^ correspond to the stretching vibrations of the C–O–C ring, indicative of epoxy groups. Additional peaks related to *cis*-1,4-polyisoprene are identified at 2956 cm^−1^, 2920 cm^−1^, and 2860 cm^−1^ (corresponding to C–H stretching vibrations), as well as at 1643 cm^−1^ (the bending of C=C) and 1440 cm^−1^ (the bending of CH_2_). These features persist in the EGA spectrum, but the disappearance of the epoxy group peak indicates ring opening of ENR's epoxy groups during the reaction. The hydroxyl band in EGA undergoes a red shift relative to GO, suggesting hydrogen bonding between hydroxyl groups from ENR and oxygen-containing groups on graphene sheets.^31,32^ As the ENR content in EGA increases, the hydroxyl band shifts further, reflecting enhanced hydrogen bonding (Fig. 3b). After PDMS coating, characteristic peaks at 2960 cm^−1^, 2918 cm^−1^, 1259 cm^−1^, 1016 cm^−1^, 864 cm^−1^ and 796 cm^−1^ emerge, corresponding to asymmetric stretching, symmetric stretching and deformation of CH_3_, Si–O stretching, CH_3_ swinging, and Si–C stretching vibrations, respectively. These confirm the successful deposition of the PDMS layer on the composite aerogel.^33^

The interaction between ENR and GO was further analyzed through X-ray diffraction (XRD) and Raman spectroscopy. In the XRD pattern (Fig. 3c), GO exhibits a characteristic diffraction peak at 2*θ* = 9.38°. Following hydrothermal treatment, reduction of oxygen-containing functional groups causes graphene sheets to restack, resulting in the GA peak shifting to 2*θ* = 26.56°.^34^ Due to its amorphous structure, ENR shows a broad diffraction peak at 2*θ* = 18.9°. EGA display graphene related peaks that shift leftward as ENR content increases, indicating reduced π–π interactions between graphene sheets and increased interlayer spacing. For EGA_80%_, the peak aligns with the broad diffraction feature of ENR, reflecting significant interaction between ENR and graphene sheets. Raman spectra (Fig. 3d) of GO, GA and EGA highlight two prominent peaks: the D peak at 1349 cm^−1^, representing disordered vibrations associated with defects in graphene, and the G peak at 1590 cm^−1^, corresponding to the in-plane C–C stretching of sp^2^ carbon atoms. The D to G intensity ratio of (*I*_D_/*I*_G_) reflects material disorder and defect density. After hydrothermal reduction, GA exhibits an increased *I*_D_/*I*_G_ ratio (0.96 to 1.14), suggesting the formation of additional sp^2^ domains.^35^ In EGA, *I*_D_/*I*_G_ ratios decreases progressively from 1.06 (EGA_20%_) to 1.02 (EGA_40%_) and 0.98 (EGA_60%_) as ENR content increases, likely due to hydrogen bond interactions between GO and ENR, improving structural order. However, the *I*_D_/*I*_G_ ratio rises to 1.08 for EGA_80%_, indicating a transition toward disorder, consistent with SEM observations of excessive ENR stacking and fusion. This behavior aligns with the XRD findings, supporting the hypothesis of increased disorder at high ENR content.

**Fig. 4:**
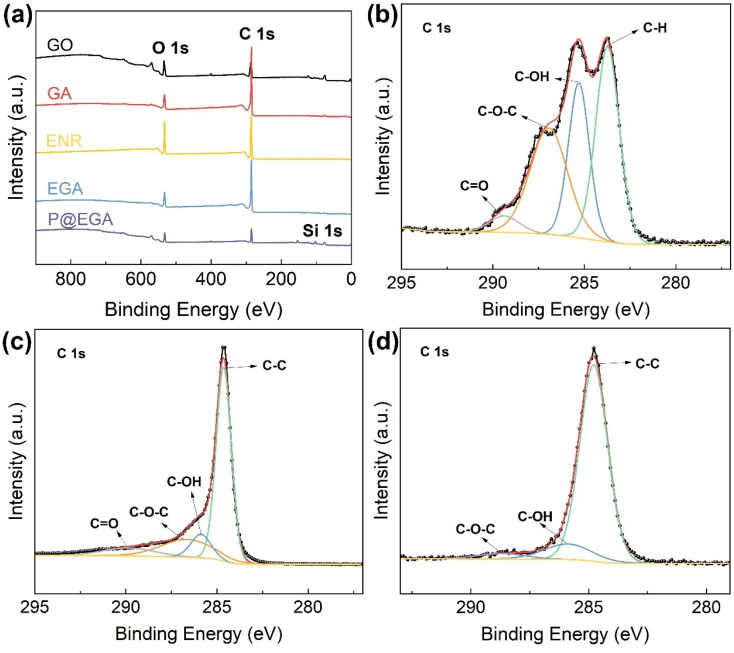
(a) XPS spectra of GO, GA, ENR, EGA and P@EGA, high resolution C 1s core level XPS spectra of (b) GO, (c) GA, and (d) P@EGA.

The changes in atomic composition and chemical structure of composite aerogels were further analyzed using X-ray photoelectron spectroscopy (XPS). The wide scan XPS spectrum in Fig. 4a revealed O 1s and C 1s peaks at 534.1 eV and 285.1 eV, respectively, for all materials. The introduction of the PDMS layer in the P@EGA was evidenced by the appearance of a new Si peak at 103.1 eV. The C 1s spectra of GO, GA and P@EGA presented in Fig. 4b–d demonstrated significant differences. The spectrum of GO deconvolved into four peaks at 284.5 eV, 285.3 eV, 286.9 eV, and 289.4 eV, corresponding to C=C, C–OH, C–O–C, and C=O, respectively, reflecting the abundance oxygen-containing functional groups. In GA, the proportions of C–OH and C–O–C decreased by 16.4% and 12.68%, respectively, due to the hydrothermal reduction that removed epoxy and hydroxyl groups from GO. In the P@EGA the C–O–C content further decreased, accompanied by a 22.64% increase in C=C. This shift indicates ring-opening of epoxy groups in GO and ENR, forming robust hydrogen bonds that enhance interfacial compatibility and structural integrity.^31,36^

### 3.3 Adsorption and separation properties of composite aerogels

**Fig. 5:**
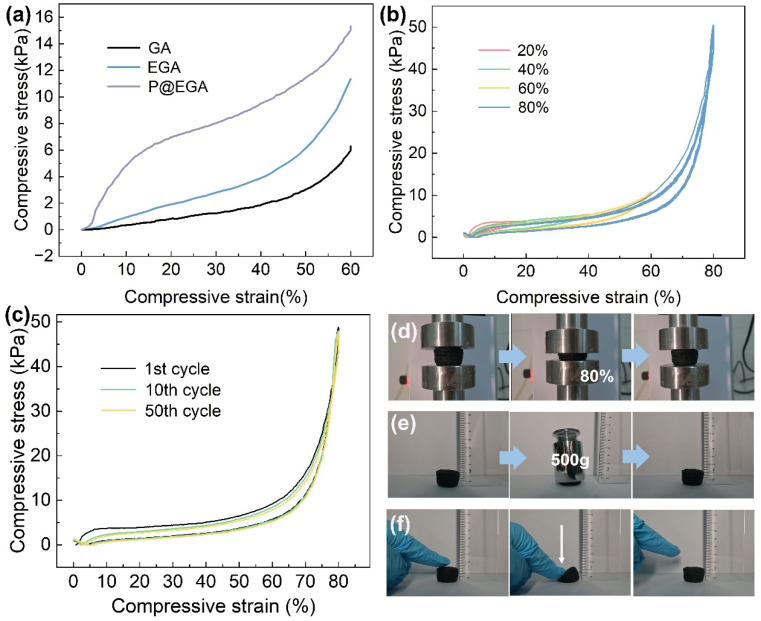
(a) Compressive stress–strain curves of GA, EGA and P@EGA; (b) stress–strain curves of P@EGA cyclic compression under different strains of 20%, 40%, 60% and 80%; (c) stress–strain curves of P@EGA under cyclic compression for 50 cycles; (d–f) digital images of various stress test processes of P@EGA.

The mechanical properties of GA, EGA and P@EGA were evaluated through compression tests. The compression stress–strain curves of these aerogels under 60% strain are illustrated in Fig. 5a. Notably, the samples exhibited a characteristic crescent-shaped stress–strain curve, which included a linear elastic region associated with the bending of the layered structure (strain less than 10%), a plastic deformation region in the intermediate range (10–40%), and a sudden increase in stress in the final stage (greater than 40%) related to structural densification. EGA's maximum stress under 60% strain was 11.37 kPa, which is approximately double that of the GA (6.29 kPa), indicating that the incorporation of ENR particles within the graphene sheets not only enhanced surface roughness but also improved the mechanical properties. Furthermore, the P@EGA demonstrated the highest stress resistance (15.33 kPa) under the same strain, suggesting that the PDMS layer providing additional flexibility and reinforcement. The fatigue resistance of the P@EGA was assessed under varying cyclic strains and cycles. Fig. 5b presents a series of cyclic compression stress–strain curves for the P@EGA under different strains of 20%, 40%, 60%, and 80%. The results indicated that there was no residual strain following the release of compression strains ranging from 20% to 80%. As the strain increased, the sample exhibited slight plastic deformation, yet remained relatively stable while transitioning between the various strain ranges. As illustrated in Fig. 5c, the P@EGA was subjected to compression for 50 cycles at an 80% strain. Following the 50th cycle, the maximum compression stress of P@EGA still reached 47.66 kPa, which retained 97.54% of its initial value, indicating remarkable compressibility. Fig. 5d–f and Movie S1† demonstrate that when the P@EGA was compressed to 80% on a universal testing machine, a weight of 500 g was applied for 10 s, along with downward pressure applied to the left side using fingers. Notably, the material exhibited no damage and was able to recover its original shape quickly. The outstanding mechanical properties and flexibility of the aerogel can be primarily attributed to the 3D network, ENR-induced hydrogen bonding and PDMS layer reinforcement.

**Fig. 6:**
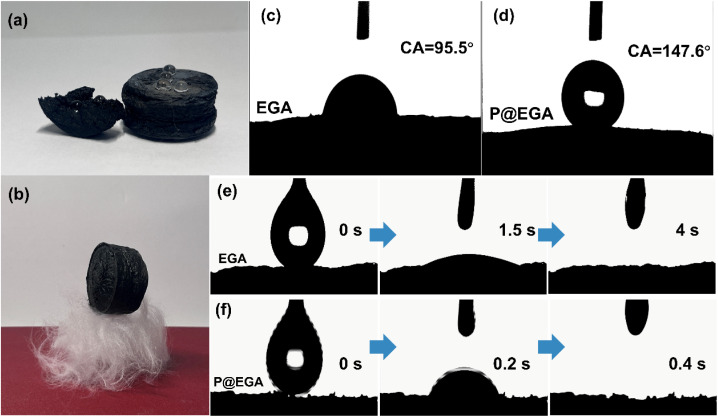
Digital images of (a) static water drops on the internal/external surface of P@EGA, (b) it can easily stand on the eiderdown cotton; the water contact angle of (c) EGA and (d) P@EGA, oil contact angle testing process of (e) EGA and (f) P@EGA.

The P@EGA demonstrates exceptional hydrophobicity and oleophilicity. It retains water droplets on its surface and within its structure, maintaining an ultralight density of 0.0104 g cm^−3^, even after PDMS coating, allowing it to rest effortlessly on down cotton (Fig. 6a and b). The water contact angle for the EGA is 95.5°, whereas the standard aerogel achieves a remarkable 147.6° (Fig. 6c and d). Diesel fuel takes 4 s to diffuse into the EGA, compared to only 0.4 s for complete adsorption in the standard aerogel (Fig. 6e, f and Movie S2†). The PDMS layer further enhances the aerogel's ability to adsorb diesel fuel entirely, underscoring its hydrophobic and lipophilic properties.

**Fig. 7:**
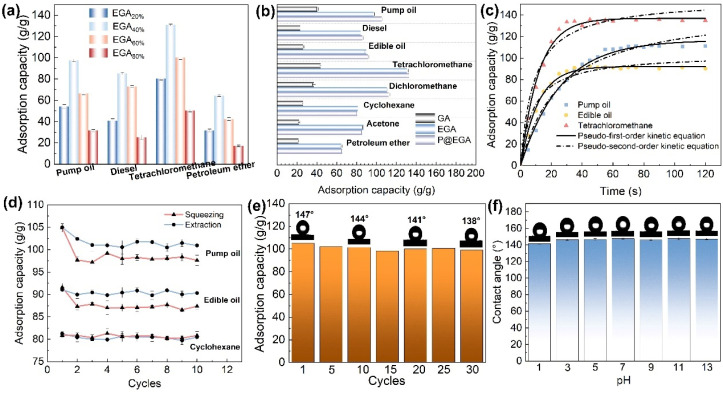
(a) Adsorption capacity of EGA with different ENR contents for various oil products and organic solvents; (b) adsorption capacity of GA, EGA and P@EGA for various oil products and organic solvents; (c) adsorption kinetics curve of three models of organism by P@EGA; (d) regeneration test of P@EGA using squeezing and extraction, respectively; (e) 30 cycles of adsorption-squeezing for pump oil by P@EGA; (f) water contact angle of P@EGA at different pH solutions.

To investigate the effect of ENR introduction on the adsorption capacity of composite aerogels, EGA with varying ENR ratios were employed for the adsorption of pump oil, diesel fuel, tetrachloromethane, and petroleum ether. The results (Fig. 7a) indicated that the adsorption capacity of EGA increased initially with the higher ENR content but declined gradually when the ENR proportion exceeded 60%. This decrease can be attributed to the increased density and aggregation of ENR particles at higher concentrations, which reduces the internal channels of the aerogel. As shown in Fig. 7b, the adsorption capacity of EGA ranged from 64.43 to 130.47 g g^−1^, significantly surpassing that of GA (21.44–43.59 g g^−1^). This improvement can be attributed to the introduction of ENR particles between the GO sheets, which significantly extends the specific surface area of the 3D network structure and provides more adsorption sites. The P@EGA exhibited a marginally higher adsorption capacity (65.37–132.75 g g^−1^) compared to EGA, indicating that hydrophobic modification enhanced water repellency without significantly altering its lipophilicity. Interestingly, the adsorption capacity of P@EGA for low-density petroleum ether was only 65.37 g g^−1^, whereas it reached 132.75 g g^−1^ for carbon tetrachloride. This suggests that the adsorption capacity of the samples is roughly proportional to the density of the adsorbate, implying that pore distribution plays a dominant role in influencing adsorption behavior.^37^

The P@EGA was immersed in three representative adsorbates, and the relationship between adsorption capacity and time was recorded and plotted (Fig. 7c). To further investigate the adsorption behavior of P@EGA, the experimental data were fitted using the pseudo-first-order kinetic model (2) and the pseudo-second-order kinetic model (3):

2 ln(*Q*_e_ − *Q*_*t*_) = *Q*_e_ − *k*_1_*t*

3 *t*/*Q*_*t*_ = 1/*k*_2_*Q*_e_ + *t*/*Q*_e_

where *Q*_e_ and *Q*_*t*_ are the adsorption capacity of the adsorbate at saturation and time *t*, respectively. *k*_1_ and *k*_2_ are the reaction rate constants of the pseudo first order model and pseudo second order model, respectively.

The analysis of the adsorption process reveals that the P@EGA demonstrates a distinct kinetic behavior across the three tested adsorbates: pump oil, edible oil, and tetrachloromethane. During the initial phase, the adsorption capacity rises rapidly, reflecting the high availability of active sites. This is followed by a gradual approach to saturation, as the aerogel's sites become increasingly occupied. The saturation times—75 s for pump oil, 45 s for edible oil, and 30 s for tetrachloromethane—underscore the influence of adsorbate properties on diffusion rates. Low-viscosity substances like tetrachloromethane diffuse more efficiently into the aerogel's porous network, achieving saturation quickly. High-viscosity substances like pump oil exhibit slower diffusion but adhere strongly to the aerogel's rough surfaces, leading to enhanced adsorption at equilibrium. The kinetic fitting results (Table 1) suggest that the adsorption process is better described by the pseudo-first-order model, with determination coefficient *R*^2^ values supporting this conclusion. This indicates a predominance of physical adsorption, driven by surface interactions and diffusion, rather than chemical bonding mechanisms. Overall, these results highlight the aerogel's versatility in adsorbing both high- and low-viscosity organics efficiently. In addition, EGA and P@EGA were immersed in two different adsorbates (pump oil and tetrachloromethane) and their adsorption capacities at various time points were recorded (ESI Fig. S3†). In the adsorption of pump oil, the adsorption rate of EGA was slightly higher than that of P@EGA, but the final adsorption capacity of both materials was comparable. There was no significant difference in the performance of the two materials during the adsorption of carbon tetrachloride. This suggests that although the impregnation of the PDMS layer may affect the rate of adsorption of highly viscous organic compounds by the aerogel, the hindrance caused is not significant.

**Table 1:** Pseudo-first-order and pseudo-second-order constants and correlation coefficients for adsorption of three adsorbates on P@EGA adsorbent

Adsorbate	Pseudo-first-order kinetic model			Pseudo-second-order kinetic model		
	*Q*_e_ (g g^−1^)	*k*_1_ (s^−1^)	*R* ^2^	*Q*_e_ (g g^−1^)	*k*_2_ (g g^−1^ s^−1^)	*R* ^2^
Pump oil	116.5976	0.03894	0.99272	147.5131	0.00026	0.97583
Edible oil	92.2327	0.08772	0.99304	103.7898	0.00118	0.95743
Tetrachloromethane	136.9812	0.08442	0.99063	155.0107	0.00075	0.95666

The regeneration ability of P@EGA has been thoroughly evaluated using three organic matter models (pump oil, edible oil, and cyclohexane) and two regeneration methods (extrusion and extraction). As shown in Fig. 7d, the adsorption capacity of the composite adsorbent decreased during the second adsorption cycle for both pump oil and edible oil when regenerated using the extrusion method. This reduction can be attributed to residual adsorbates trapped within aerogel structure after extrusion. Notably, higher viscosity organic matter resulted in greater residue retention. Despite this, the extrusion-regenerated composite adsorbent achieved recovery rates of 93.1% and 95.4% for pump oil and edible oil, respectively, after 10 adsorption cycles. In contrast, the extraction method exhibited superior performance, with almost no loss of adsorption capacity for either pump oil or edible oil across 10 cycles. Furthermore, the recovery rate for cyclohexane exceeded 99% after 10 cycles of adsorption regeneration, regardless of the method employed. This exceptional recovery is primarily due to the volatile nature of cyclohexane, which prevents significant retention within the aerogel structure. Although the extraction method is more effective for desorbing organic matter, it incurs higher costs and requires more time compared to the extrusion method. Based on these findings, we recommend prioritizing the low-cost and high-efficiency extrusion regeneration method. The P@EGA demonstrated excellent reusability through simple extrusion. SEM images of the P@EGA after the tenth adsorption–desorption cycle (ESI Fig. S4†) revealed well-preserved layered pores and ENR particles dispersed within the sheets, indicating the recycled aerogel retained its original structure and shape. As shown in Fig. 7e, the measured contact angle of 138° after 30 cycles confirmed minimal loss of the PDMS layer. This further demonstrated that the dual enhancement effect of ENR and PDMS on the composite aerogels remained effective, ensuring good stability even after multiple regeneration cycles. In addition, HCl and NaOH solutions with different pH values were prepared as test liquids to evaluate the hydrophobic stability of P@EGA (Fig. 7f).^38^ The results indicate that the water contact angle of P@EGA remains relatively stable across a broad spectrum of pH values, demonstrating high hydrophobicity (CA = 140.5°) even at the lowest pH level (pH = 1). This characteristic renders it suitable for use in diverse aqueous environments. Table 2 provides a comparative analysis of various adsorbents in terms of adsorption capacity, adsorption rate, and recycling performance. Notably, the P@EGA in this study outperformed many other adsorbents, showcasing distinct advantages for water–oil separation applications.

**Table 2:** Comparison of adsorption properties of various absorbents

Adsorbents	Density (mg cm^−3^)	Contact angle	Compressibility	Adsorbates	Adsorption capacity (g g^−1^)	Regeneration	Reference
Superhydrophobic cotton	—	156°	—	Oils, organic solvents	20–50	Vacuum filtration	39
NiCo@rGO aerogel microsphere	7.2	130°	—	Oils, organic solvents	107–270	Solvent extraction	9
PDMS sponge	—	100°–143°	—	Oils, organic solvents	1–8	Squeezing	40
Biomass carbon aerogel	48	135°	—	Oils, organic solvents	16–50	Distillation	41
Carbon aerogel	0.16	—	The stress retention rate at 50% strain was 88% after 1000 cycles	Organic solvents	215–913	Heating	42
Reduced graphene aerogel	Low density	150.51°	—	Oils, organic solvents	19–26	Burning/Distillation	43
Nanocellulose/graphene aerogel	18	130°	The stress retention rate at 90% strain was 98% after 100 cycles	Oils, organic solvents	25–58	Squeezing	44
Magnetic MCS/tof aerogel	Ultralight	153°	The stress retention rate at 70% strain was 89% after 10 cycles	Oils, organic solvents	37.1–88.4	Squeezing	45
PDMS/CB@PU sponge	—	155.4°	No significant change in strength at 50% strain after 500 cycles	Oils, organic solvents	28.5–68.7	Squeezing	46
P@EGA	10.4	147.6°	The stress retention rate at 80% strain was 97.5% after 50 cycles	Oils, organic solvents	65.3–132.7	Squeezing/Solvent extraction	This work

**Fig. 8:**
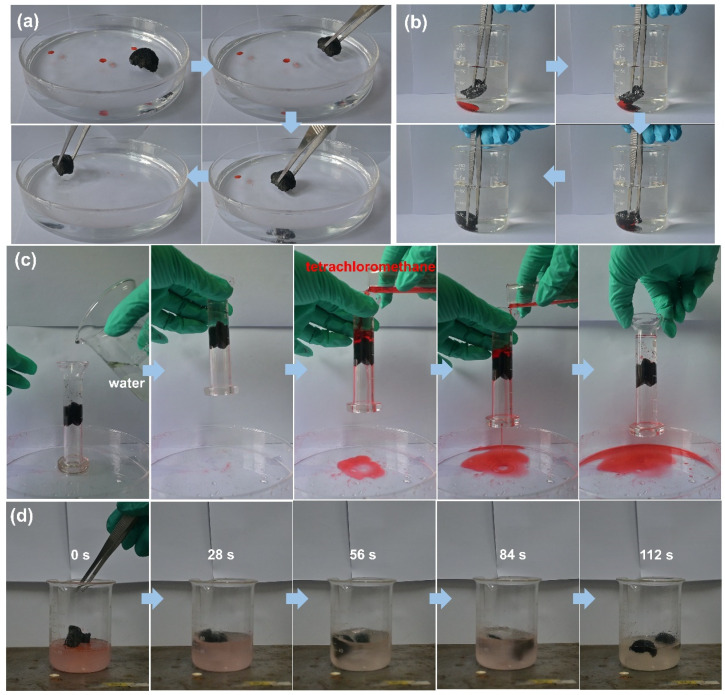
The adsorption process of (a) diesel and (b) tetrachloromethane by P@EGA in water; (c) separation process of water–oil mixture driven by gravity and (d) dynamic separation process of oil in water system.

**Fig. 9:**
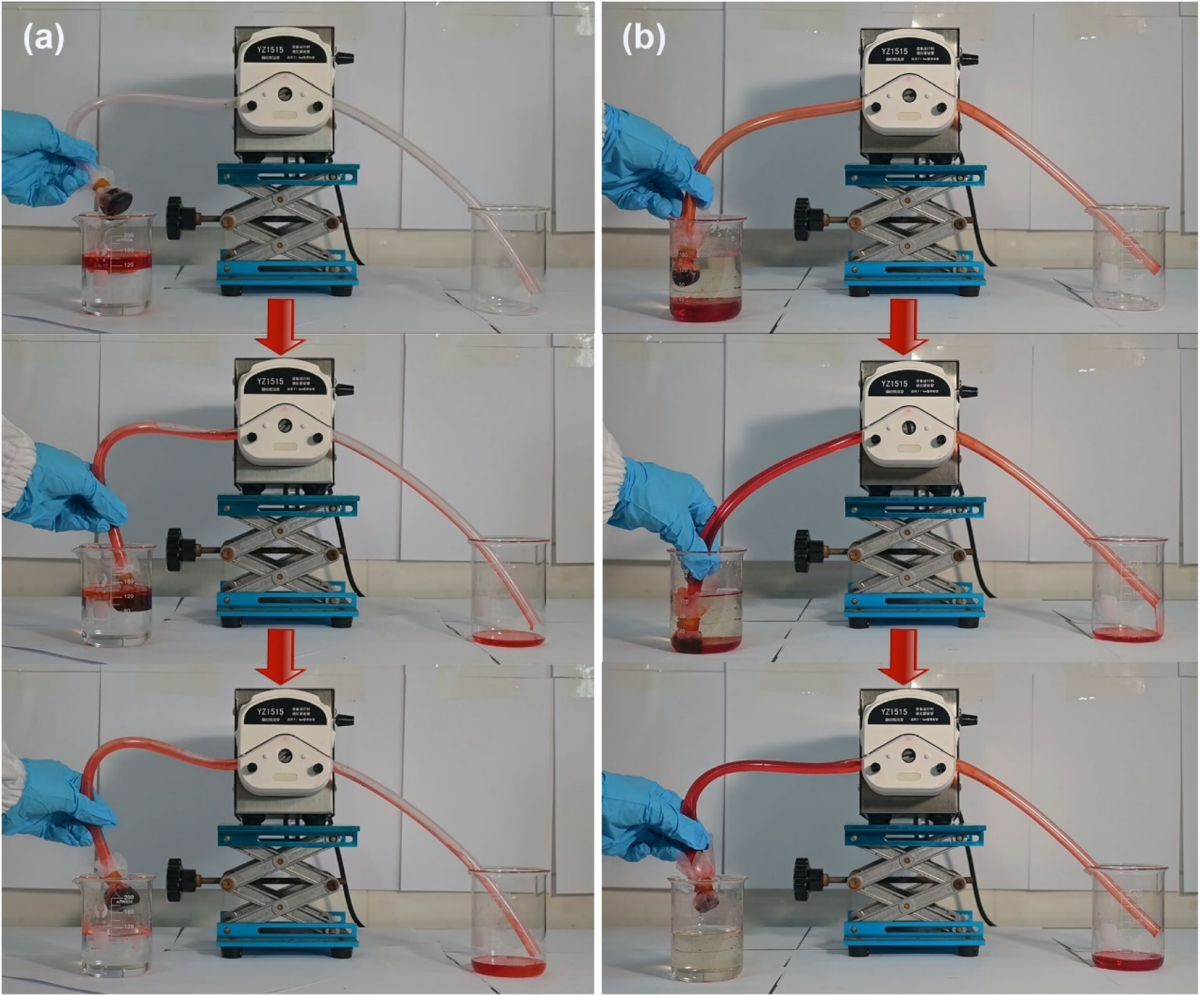
Dynamic continuous adsorption of (a) diesel oil and (b) tetrachloromethane by P@EGA and peristaltic pump.

Diesel oil and tetrachloromethane, both dyed with Sudan Red III, were utilized as model organics pollutants to assess the water–oil selectivity of the P@EGA. When the aerogel was immersed in a water–oil mixture (Fig. 8a, b and Movie S3†), it effectively and rapidly adsorbed diesel floating on the water surface and tetrachloromethane sinking to the bottom, leaving no observable residue. Upon submersion and subsequent pressing underwater, the aerogel's surface exhibited distinct phosphorescence, a phenomenon caused by an air layer trapped between the PDMS layer and the water. This is a characteristic feature of hydrophobic surfaces. Fig. 8c and Movie S4† illustrate the water–oil separation capability of the P@EGA under gravity-driven conditions. Due to its hydrophobic properties, water could not penetrate the aerogel, while tetrachloromethane readily passed through a glass tube. The upper water level remained constant throughout the experiment. The water–oil separation efficiency of P@EGA was determined to be as high as 98%, calculated as the ratio of the weight of tetrachloromethane collected to the weight of tetrachloromethane initially added to the mixture.^47^ To further simulate practical scenarios of oil-in-water contamination, an oil-in-water emulsion containing dyed diesel oil was prepared using high-speed stirring. The composite aerogel was employed for dynamic adsorption and separation (Fig. 8d and Movie S5†). After 112 s of stirring, the adsorption process was complete, leaving no visible residual red oil in the container. The dynamic collection of diesel oil floating on the surface of the water and tetrachloromethane sinking underwater was conducted separately using a peristaltic pump with P@EGA as the filter material (Fig. 9 and Movie S6†). The results showed that even when P@EGA was inserted underwater, it only selectively absorbed oil but not water. At the conclusion of the collection process, the water in the beaker on the left remained unchanged, whereas the beaker on the right contained only oil, devoid of any water admixture. This illustrates the exceptional water–oil separation capability of P@EGA.

## 4 Conclusions

In this study, we successfully developed a PDMS-coated epoxidized natural rubber–graphene composite aerogel (P@EGA) through the incorporation of ENR particles and PDMS. This approach provided dual reinforcement to graphene aerogels in an eco-friendly and straightforward manner. Comprehensive characterizations confirmed the effective embedding of ENR within the graphene sheets, forming hydrogen bonds that increased interlayer spacing, reduced sheet stacking, and addressed the brittleness commonly associated with 3D graphene aerogels. These improvements resulted in a stable 3D porous network. The P@EGA exhibited exceptional properties, including a low density of 0.0104 g cm^−3^, a high specific surface area of 482.362 m^2^ g^−1^ (the highest reported thus so far), remarkable hydrophobicity (contact angle = 147.6°) and robust mechanical strength (withstanding 50 kPa at 80% strain). Adsorption tests revealed its ability to adsorb various oils and organic solvents at capacities ranging from 65.37 to 132.75 g g^−1^, following a quasi-first-order kinetic model. Additionally, the aerogel could be regenerated easily *via* extrusion. The P@EGA demonstrated excellent water–oil separation capabilities under both gravity-driven and dynamic conditions, achieving continuous and efficient separation with mechanical assistance. These results highlight its significant potential for applications in water–oil separation and oil spill remediation, offering valuable insights into the development of graphene composite aerogels with enhanced mechanical properties.

## Data availability

The datasets generated and analyzed in this current study are available from the corresponding author upon reasonable request.

## Author contributions

Zirong Luo: writing – original draft, review & editing, visualization, software, formal analysis, data curation. Shenbo Huang: writing – review & editing, resources, methodology, investigation. Na Kong: software, methodology, supervision. Jizhen Zhang: writing – review & editing, supervision. Jinlong Tao: writing – review & editing, project administration, funding acquisition. Jihua Li: methodology. Shuang Li: software, resources.

## Conflicts of interest

The authors declare that they have no known competing financial interests or personal relationships that could have appeared to influence the work reported in this paper.

## Supplementary Material

RA-015-D4RA06747A-s001

RA-015-D4RA06747A-s002

RA-015-D4RA06747A-s003

RA-015-D4RA06747A-s004

RA-015-D4RA06747A-s005

RA-015-D4RA06747A-s006

RA-015-D4RA06747A-s007
