# Supplementary material for: Hydrophobic dual-polymer–reinforced graphene composite aerogel for efficient water–oil separation
Source: RSC Adv. 2025 Jan 3;15(1):1–13. doi: 10.1039/d4ra06747a (PMC11698129; doi:10.1039/d4ra06747a)
Supplement: RA-015-D4RA06747A-s007 [file RA-015-D4RA06747A-s007.pdf]

Supplementary Information (SI) for RSC Advances.
This journal is © The Royal Society of Chemistry 2024

Electronic Supplementary Information

Hydrophobic dual-polymer-reinforced graphene composite aerogel for efficient water-oil separation

Zirong Luo[1,*], Shenbo Huang[2], Na Kong[3], Jizhen Zhang[4], Jinlong Tao[1,*], Jihua Li[5], Shuang Li[1]

[1] Hainan Provincial Key Laboratory of Natural Rubber Processing, Agricultural Products Processing Research Institute, Chinese Academy of Tropical Agricultural Sciences, Zhanjiang 524001, P. R. China

[2] Guangdong Engineering & Technology Research Centre of Graphene-like Materials and Products, Department of Chemistry, College of Chemistry and Materials Science, Jinan University, Guangzhou 510632, P. R. China

[3] School of Life and Environmental Science, Centre for Sustainable Bioproducts, Deakin University, Geelong, Victoria 3216, Australia

[4] Institute for Frontier Materials, Deakin University, Geelong, Victoria 3216, Australia

[5] Chinese Academy of Tropical Agricultural Sciences, Haikou 571101, P. R. China

* Corresponding author.

E-mail address: luozirong3@163.com (Z.R. Luo), jinlongt1983@163.com (J.L. Tao)

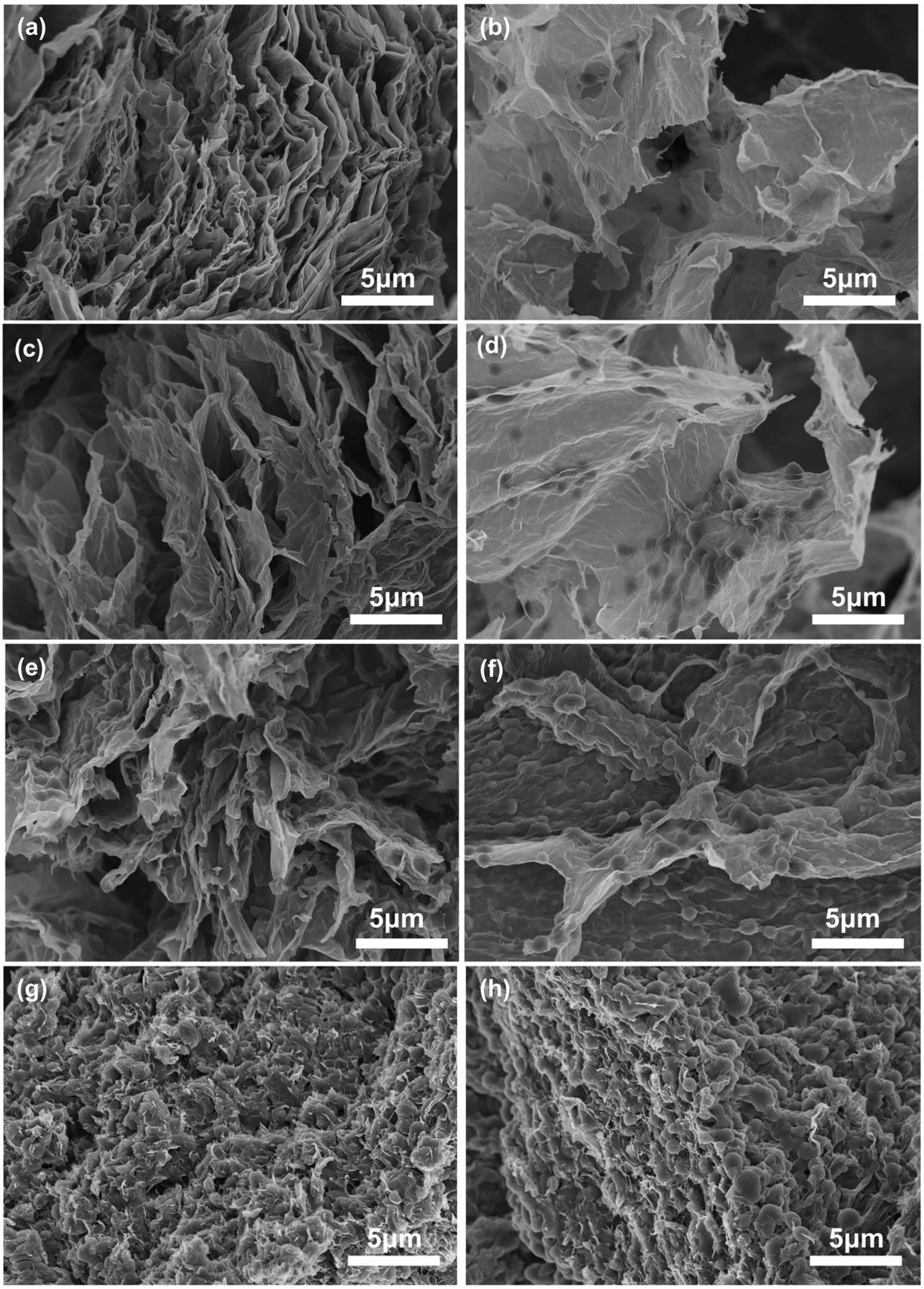

Fig. S1. SEM images of (a-b) $EGA_{20\%}$, (c-d) $EGA_{40\%}$ (e-f) $EGA_{60\%}$, (g-h) $EGA_{80\%}$ aerogel.

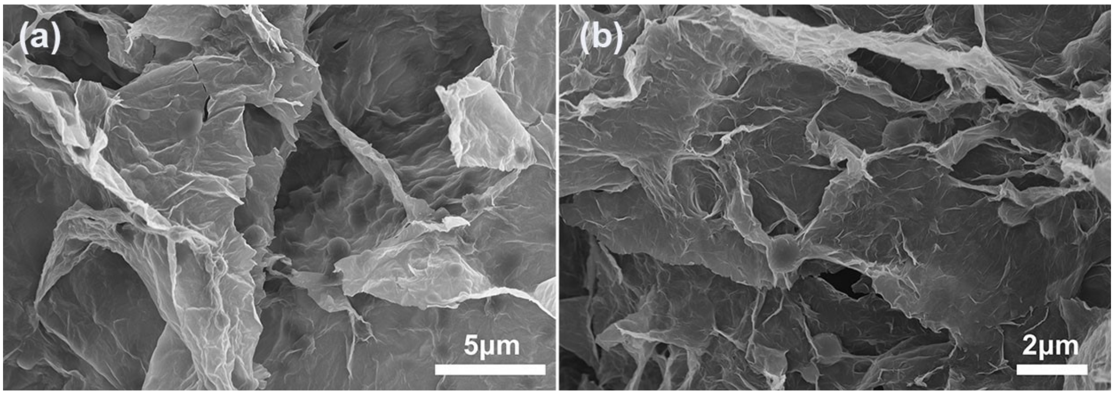

Fig. S2. (a-b) SEM images of external surface of P@EGA.

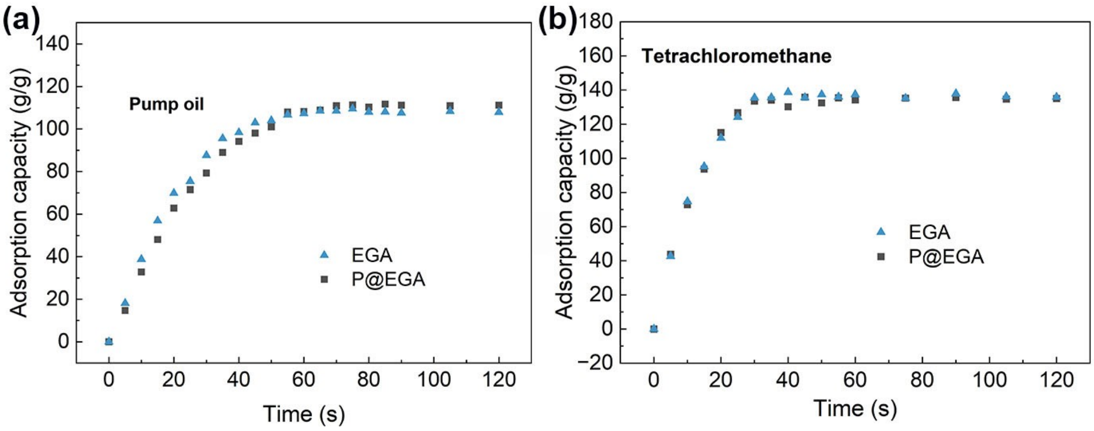

Fig. S3. Adsorption capacity of EGA and P@EGA for (a) pump oil and (b) tetrachloromethane at different adsorption time intervals..

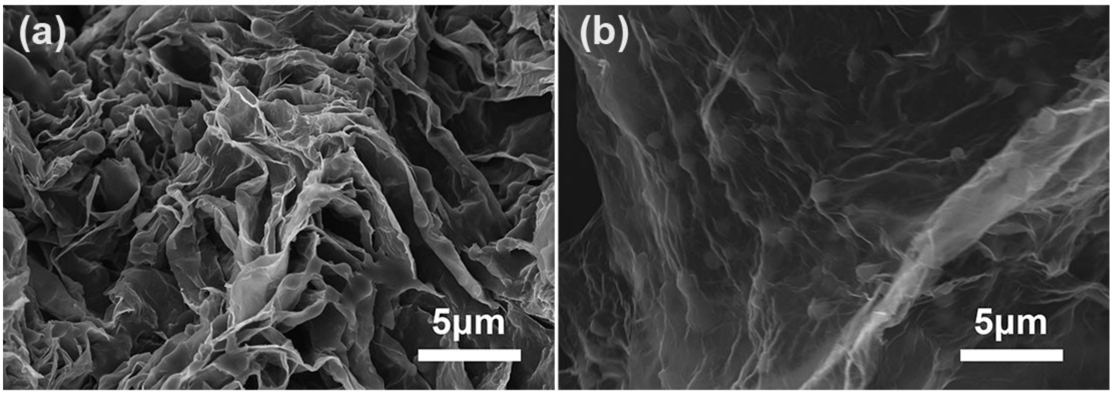

Fig. S4. (a-b) SEM images of P@EGA aerogel after the tenth cycles of adsorption-

desorption.
